# Balanced crystalloid versus saline for resuscitation in adult sepsis: a systematic review and meta-analysis

**DOI:** 10.3389/fmed.2026.1906761

**Published:** 2026-08-21

**Authors:** Lipeng Liu, Binrong Cai, Bin He

**Affiliations:** 1 Department of Emergency Medicine, West China Hospital, Sichuan University, Chengdu, China; 2 Department of Emergency Medicine, West China Tianfu Hospital, Sichuan University, Chengdu, Sichuan, China

**Keywords:** balanced crystalloids, normal saline, Plasma-Lyte, Ringer’s lactated, sepsis

## Abstract

**Background:**

Fluid resuscitation is the cornerstone of sepsis management, however, the choice between balanced crystalloid solutions and normal saline remains debated. This meta-analysis aimed to compare the efficacy of balanced solutions versus saline for fluid resuscitation in adult patients with sepsis.

**Methods:**

A systematic literature search was performed in PubMed, Embase, Scopus, and other relevant sources. Retrieved studies were rigorously screened according to predefined inclusion and exclusion criteria. Data from the included studies were extracted for analysis. The primary outcome was defined as mortality rate. Secondary outcomes included acute kidney injury (AKI), hyperchloremia, renal replacement therapy (RRT), ICU-free days, ventilator-free days, vasopressor-free days. The study protocol was registered with PROSPERO (CRD420261368142).

**Results:**

A total of eight studies (8,173 patients; 4,056 assigned to balanced solutions, 4,117 to saline) met the inclusion criteria. In the primary analysis restricted to seven randomized controlled trials, balanced solutions were associated with significantly lower mortality than saline (RR 0.92; 95% CI 0.86–0.98; *P* = 0.01; I^2^ = 37%), and a sensitivity analysis incorporating a *post-hoc* observational study yielded consistent results (RR 0.91; 95% CI 0.85–0.97). Balanced solutions were also associated with lower incidences of AKI (RR 0.86; 95% CI 0.78–0.96; *P* = 0.004), hyperchloremia (RR 0.68; 95% CI 0.52–0.90; *P* = 0.007), and RRT requirement (RR 0.74; 95% CI 0.56–1.00; *P* = 0.05), and with a greater number of ICU-free, ventilator-free, and vasopressor-free days (all *P* < 0.05).

**Conclusion:**

In adult patients with sepsis, treatment with balanced crystalloid solutions for fluid resuscitation significantly reduces mortality (moderate certainty), decreases kidney-related adverse events, and better maintains electrolyte balance, thereby improving patient outcomes. These findings support the preferential use of balanced crystalloids over saline in this population.

**Systematic review registration:**

https://www.crd.york.ac.uk/PROSPERO/view/CRD420261368142, identifier CRD420261368142.

## Introduction

Sepsis is a life-threatening organ dysfunction caused by a dysregulated host response to infection, representing one of the leading causes of mortality among critically ill patients worldwide. According to the latest data from The Lancet, there are approximately 49 million new cases annually, with associated deaths reaching 11 million, accounting for about 20% of global mortality (1). During the pathophysiological process of sepsis, impaired tissue perfusion due to relative or absolute deficiency in effective circulating blood volume constitutes a central aspect (2). Without prompt intervention, this pathological state can be life-threatening. Therefore, early, rapid, and adequate fluid resuscitation is recognized as critical for improving patient outcomes.

Fluid resuscitation increases intravascular volume, boosts cardiac output, and improves tissue perfusion; this physiological mechanism serves as the fundamental cornerstone for achieving effective treatment (3). The 2026 Surviving Sepsis Campaign Guidelines recommend crystalloid solutions as the preferred choice for initial resuscitation (4). Currently, crystalloid solutions used in fluid resuscitation are primarily categorized into two main types: normal saline (0.9% NaCl) and balanced solutions (such as Lactated Ringer’s solution, Plasma-Lyte, etc.). Saline has long been the preferred crystalloid solution due to its low cost, wide availability, and isotonic properties with blood (5). However, its chloride ion concentration (154 mmol/L) far exceeds that of plasma (approximately 103 mmol/L). Large-volume infusion can induce hyperchloremic metabolic acidosis and may worsen acute kidney injury (AKI) through mechanisms such as renal vasoconstriction and reduced glomerular filtration rate (6, 7). Additionally, large-volume saline infusion has been shown to stimulate the release of pro-inflammatory cytokines such as IL-6 and TNF-α, potentially impairing immune function (8–10). In contrast, balanced solutions possess an electrolyte composition more closely resembling plasma extracellular fluid, demonstrating advantages in maintaining acid-base balance and protecting renal function (11, 12). Balanced solutions also inhibit the release of pro-inflammatory cytokines while promoting improvement in organ function (13, 14). Thus, the choice of crystalloid solution impacts the prognosis of patients with sepsis.

Several recent studies have compared balanced crystalloids with saline in critically ill patients, including sepsis cohorts, but have yielded inconsistent conclusions. Some studies indicate that balanced solutions may reduce mortality rate and the incidence of acute kidney injury in patients with sepsis (15, 16), while others observed no significant differences (17, 18). To address this clinical controversy, an updated appraisal of the optimal fluid resuscitation strategy is warranted, integrating the most recent evidence from randomized controlled trials.

## Methods

This systematic review and meta-analysis of randomized controlled trials compare balanced crystalloids with 0.9% saline in adult sepsis patients, assessing their respective effects on mortality, renal function, and other critical outcomes. The study was prospectively registered with PROSPERO (CRD420261368142) and is reported in accordance with the Preferred Reporting Items for Systematic Reviews and Meta-Analyses (PRISMA) guidelines.

### Data sources and search strategy

Literature published from database inception to March 1, 2026, was searched across PubMed, Embase, Scopus. To identify further relevant studies, we also performed supplementary searches by manually checking the reference lists of included articles and through web-based recommendation tools. Unpublished clinical trials were searched via the ClinicalTrials.gov website. The search strategy comprised: (sepsis OR “septic shock” OR “critically ill patients”) AND (“balanced crystalloid” OR “lactated ringer” OR “plasma-Lyte” OR “normal saline” OR “0.9% saline” OR “fluid resuscitation”) AND (clinical trial OR “random” OR “RCT”). To ensure a more comprehensive literature search, reference lists from original studies were also screened.

### Inclusion criteria

Study inclusion criteria were: ① Study type: Randomized controlled trial (RCT) or secondary analysis of RCT that compared balanced crystalloids versus normal saline for fluid resuscitation in adult sepsis patients, with reported outcomes of interest; ② Study subjects: Patients aged ≥18 years with sepsis, with or without shock; sepsis definitions could differ across studies. ③ Interventions: Studies comparing normal saline versus balanced solutions that reported outcomes of interest. Exclusion criteria were: ① Pediatric sepsis patients or pregnant women; ② Systematic reviews; ③ Retrospective observational studies (non-RCT); ④ Case reports; ⑤ Animal studies; ⑥ Conference abstracts; ⑦ Studies with unavailable full text; ⑧ Secondary analyses of RCTs that did not adjust for potential confounders in the fluid-type comparison. When multiple articles reported overlapping patient cohorts, the literature with the largest sample size, longest follow-up duration, or most complete data was preferentially selected for analysis of specific outcomes.

### Study selection

Two investigators independently identified and reviewed relevant literature according to the inclusion and exclusion criteria. Any discrepancies during the screening process were resolved through careful deliberation by a third investigator.

### Data extraction

The researchers collaboratively designed a data collection form, including first author, publication year, study design, study population, demographic characteristics, and outcomes of interest. Two investigators independently extracted data from each study. Discrepancies were reviewed by a third investigator. For large RCTs that reported sepsis-subgroup outcomes but did not provide baseline characteristics for that subgroup separately, we extracted the baseline data from the entire cohort as a pragmatic substitute. Outcome data were still extracted exclusively for the sepsis subgroup.

### Risk of bias

The quality of the included randomized controlled trials was assessed using the risk of bias assessment tool (RoB 1) recommended in the Cochrane Handbook for Systematic Reviews (19). The assessment was conducted independently by two researchers, with disagreements resolved through consensus.

### Clinical outcomes

The primary outcome was the 30- or 90-days mortality. Mortality rates reported in the articles were collected. When multiple mortality data points were reported in an article, the longest follow-up period was selected for analysis; if unavailable, in-hospital mortality was reported. Secondary outcomes included acute kidney injury (AKI), hyperchloremia, requirement for renal replacement therapy (RRT), ICU-free days, ventilator-free days, vasopressor-free days. AKI was defined as a short-term increase in serum creatinine by ≥0.3 mg/dL or a ≥1.5-fold increase in creatinine levels according to KDIGO criteria (20). Hyperchloremia was defined as serum chloride levels ≥110 mmol/L. ICU-free days, ventilator-free days, vasopressor-free days refer to the number of days alive and free from the specified therapy in the first 28 days after enrollment. Length of hospital stay and duration of RRT were not analyzed due to heterogeneity in reporting among the included studies.

### Statistical analysis

Risk ratios (RR) with 95% confidence intervals (CI) were used for categorical endpoints, and mean differences (MD) were used for continuous outcomes. A *p*-value < 0.05 was considered statistically significant. Heterogeneity was assessed using the I^2^ statistic, with significance considered when I^2^ > 50%. A fixed-effects model was initially used; when I^2^ > 50%, a random-effects model was applied. Where necessary, medians and interquartile ranges were converted to means and standard deviations (21). Obviously skewed data were excluded from outcome analysis. Sensitivity analysis was conducted using the leave-one-out meta-analysis. Statistical analyses were performed using Review Manager (Version 5.4) and R 4.2.1. The quality of evidence for the outcomes was evaluated using the grading of recommendations, assessment, development and evaluation (GRADE) approach (22). For the primary analysis, only RCTs with direct randomized comparisons were included in the primary analysis. *Post-hoc* observational analyses from RCT cohorts were excluded from the primary analysis but included in sensitivity analyses if adjusted for confounders.

## Results

### Literature search results and characteristics

**FIGURE 1:**
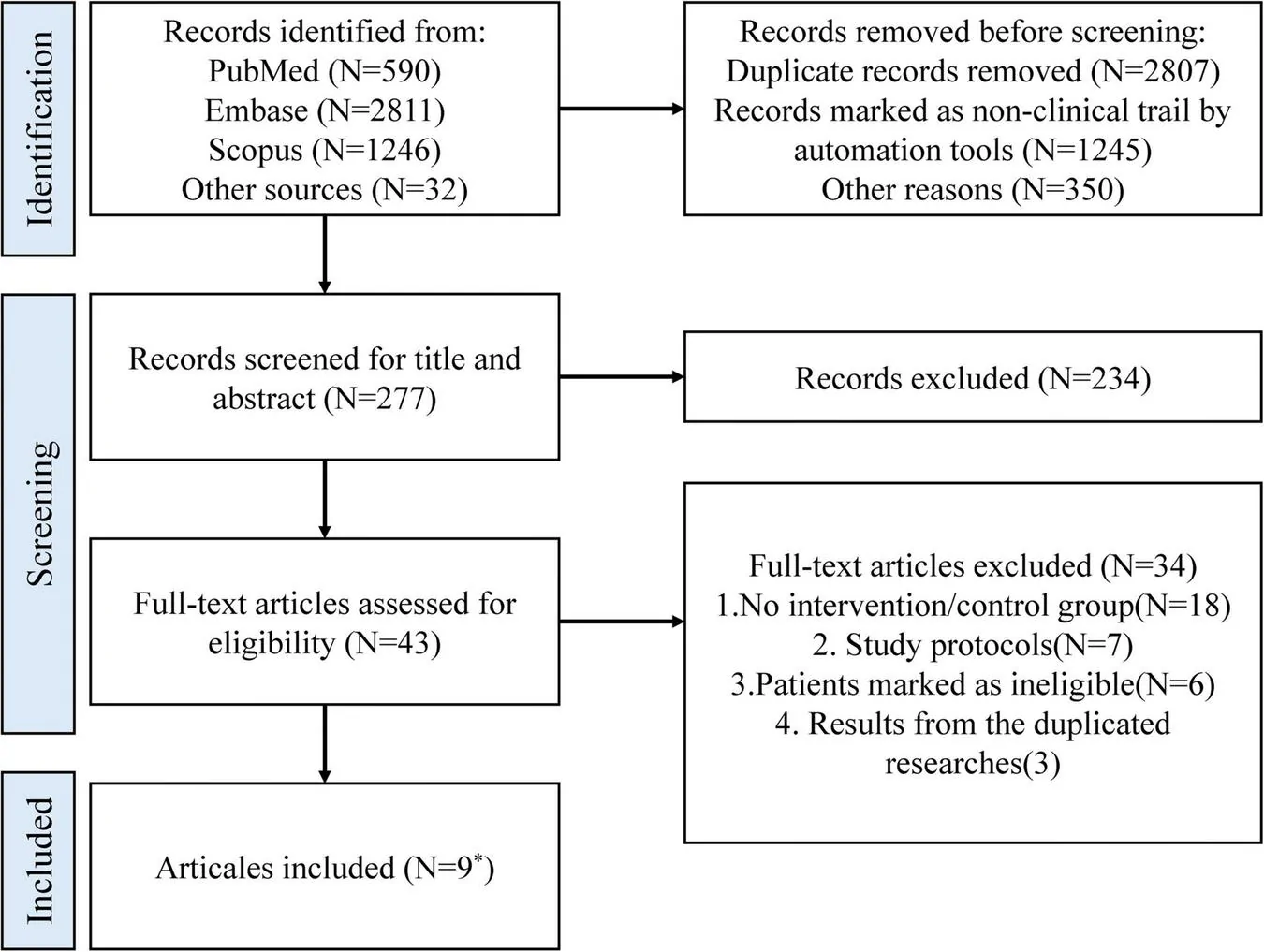
The selection flow diagram. *: There are two articles originating from the same clinical trial but reporting different outcome measures; hence, two publications were included for specific outcome analyses.

The study selection process is shown in Figure 1. A total of 4,679 records were identified. After removing duplicates, non-clinical trial records, and other exclusions, 277 records underwent title/abstract screening, of which 234 were excluded. The remaining 43 full text articles for eligibility assessment. During full-text review, 34 articles were excluded for the following reasons: no intervention or control group (*n* = 18), study protocols (*n* = 7), ineligible patient population (*n* = 6), and duplicate reports of the same research (*n* = 3). Ultimately, 9 articles were included (15, 23–30). Of note, these 9 articles correspond to 8 independent clinical studies: seven are primary reports of randomized controlled trials (RCTs) (15, 24–30), one is a *post-hoc* observational analysis of the CLOVERS trial cohort (23), and one is a secondary analysis of the SMART trial (29) reporting outcomes not covered in the primary publication (15).

The eight studies included a total of 8,173 sepsis patients, of whom 4,056 were allocated to the balanced solutions group (BS) and 4,117 to the saline group (NS) (15. 23–30). The baseline characteristics of patients across the included cohorts, including age, gender, and SOFA scores, showed no statistically significant differences between the two groups (Table 1). The balanced solutions used in the BS comprised Lactated Ringer’s solution, Ringer’s acetate and Plasma-Lyte. Specifically, three studies utilized Lactated Ringer’s solution alone, one study utilized Ringer’s acetate, three studies employed Plasma-Lyte 148 alone, and one study used either Lactated Ringer’s solution or Plasma-Lyte (Supplementary Table 1). The risk of bias was assessed using the Cochrane Collaboration’s tool, with results presented in Supplementary Figure 1.

**TABLE 1:** Characteristics of included studies.

References	Sepsis criteria	Setting	Population	Overall trial population BS/NS	Sepsis cohort analyzed BS group (*N*)	Sepsis cohort analyzed NS group (*N*)	Country	Other
Young et al. (30)	APACHE diagnostic codes	Multicenter, Double-blind, cluster randomized, double-crossover trial	Patients in the ICU	1152/1110^£^	Plasma-Lyte148 (41)	0.9% Saline (43)	Australian and New Zealand	SPLIT trail ACTRN12613001370796
Semler et al. (15)*	ICD-10-CM	Multicenter, a pragmatic, unblinded, cluster-randomized, multiple-crossover trial	Critically ill adults	7942/7860^£^	Lactated Ringer’s or Plasma-Lyte A (1167)	0.9% Saline (1169)	America	SMART trail NCT02444988 and NCT02547779
Brown et al. (29)*	ICD-10-CM	Multicenter	Sepsis	824/817	Lactated Ringer’s solution or Plasma-Lyte A (824)	0.9% saline (817)	America	A secondary analysis of the SMART trail NCT02444988
Golla et al. (28)	Sepsis-3	Single center, open-label RCT	Sepsis	80/80	Ringer’s lactate (80)	0.9% saline (80)	India	–
Pagano et al. (27)	Sepsis-3	Multicenter, randomized open-label clinical trial	sepsis and septic shock	35/49	Ringer’s lactate (35)	0.9% saline (49)	Italy	–
Zampieri et al. (26)	Sepsis-3	Multicenter, Double-blind, factorial, randomized clinical trial	Critically ill patients	5230/5290^£^	Plasma-Lyte148 (966)	0.9% saline (1015)	Brazil	BaSICS trial NCT02875873
Finfer et al. (25)	Sepsis-3	Multicenter, double-blind, randomized, controlled trial	Critically ill adults	2515/2522^£^	Plasma-Lyte 148 (1068)	Saline (1026)	Australian and New Zealand	NCT02721654
Zhang et al. (24)	Sepsis-3	Single center, unblinded, and parallel-controlled trial	Sepsis	73/43	Ringer’s acetate solution (73)	Saline (43)	China	NCT03685214
Gelbenegger et al. (23)	–^#^	Multicenter, unblinded, randomly	Sepsis-induced hypotension	622/690	Ringer’s lactated (622)	0.9% saline (690)	U.S.	A secondary analysis of the CLOVERS trial

*These two articles originating from the same clinical trial but reporting different outcome measures; hence, two publications were included for specific outcome analyses. ^£^Data in the entire study population, not just in patients with sepsis cohort. ^#^In the CLOVERS trial, sepsis was diagnosed based on the clinical criteria of suspected or confirmed infection accompanied by sepsis-induced hypotension. BS, balanced solutions group. NS, saline group.

### Mortality

**FIGURE 2:**
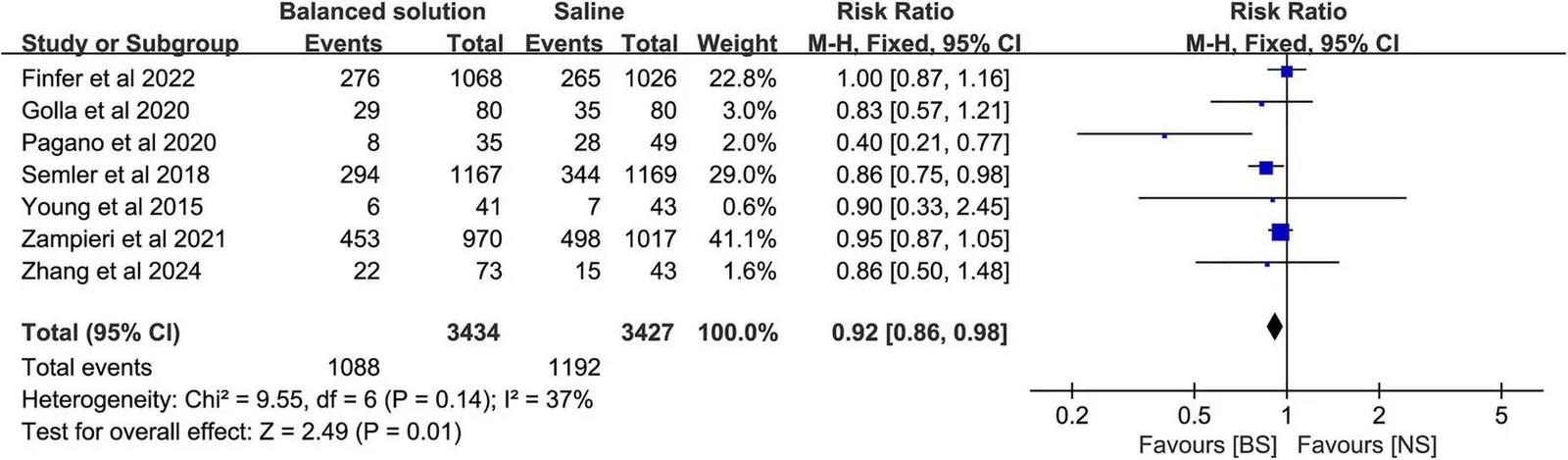
Forest plot of mortality in the primary RCT-only meta-analysis. BS, balanced crystalloids; NS, normal saline.

For the primary analysis, we restricted the pooled population to the seven RCTs that provided direct randomized comparisons between balanced crystalloids and normal saline. Pooling data from these seven RCTs showed a mortality rate of 34.8% (1192/3427) in the NS group and 31.7% (1088/3434) in the BS group (15, 24–30). Compared with the NS group, the BS group demonstrated a statistically significant reduction in overall mortality (RR 0.92; 95% CI 0.86–0.98; *P* = 0.01), with low heterogeneity across trials (I^2^ = 37%) (Figure 2).

**FIGURE 3:**
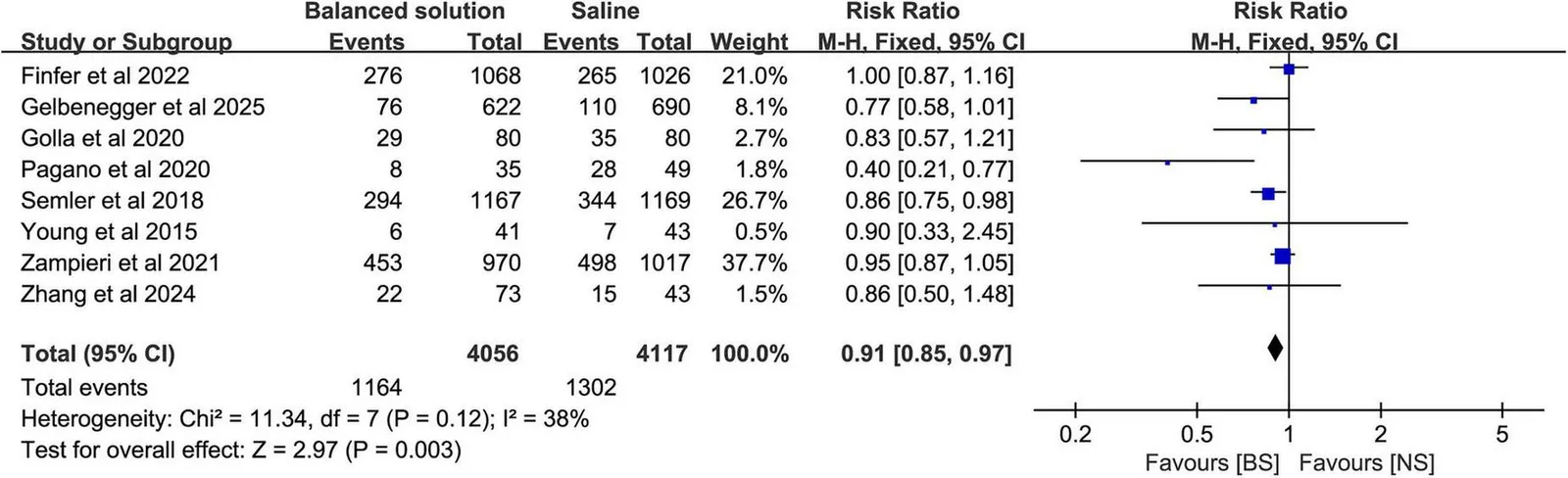
Sensitivity analysis for mortality including the *post-hoc* observational data. BS, balanced crystalloids; NS, normal saline.

We performed two prespecified sensitivity analyses. Firstly, to evaluate the impact of study design on the pooled estimate, we expanded the pooled population to include the *post-hoc* observational analysis of the CLOVERS trial cohort (23). The resulting estimate (RR 0.91; 95% CI 0.85–0.97; *P* = 0.003; I^2^ = 38%) (Figure 3) was similar to that of the primary analysis, indicating that the overall effect estimate was not substantially modified by the inclusion of this non-randomized dataset. Secondly, we observed that heterogeneity in the primary analysis originated from the Pagano (27) study. Exclusion of this study and re-analysis of the remaining six RCTs reduced heterogeneity to I^2^ = 0% while maintaining a statistically significant overall effect (RR 0.92; 95% CI 0.86–0.98).

### The incidence of AKI

**FIGURE 4:**
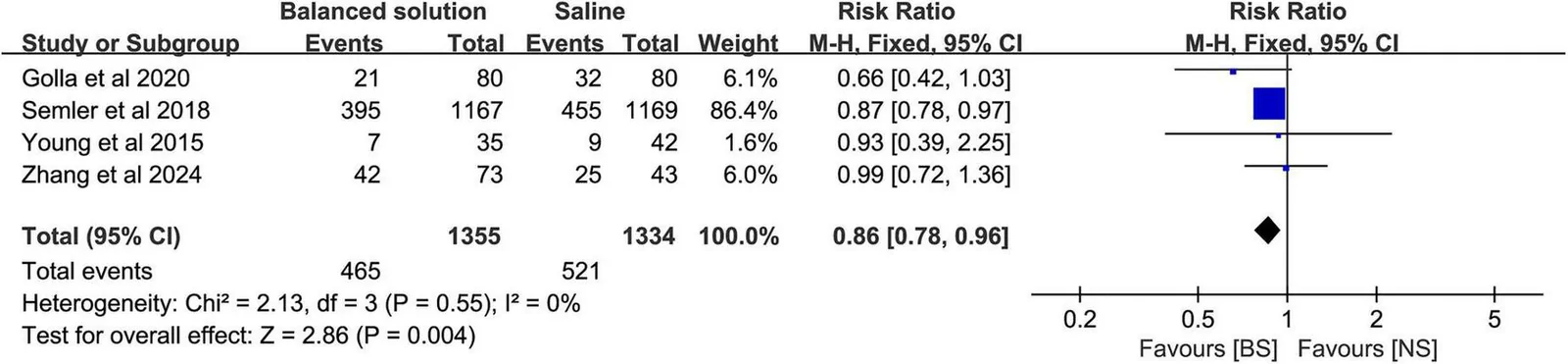
Forest plot of the incidence of acute kidney injury (AKI) in the primary RCT-only meta-analysis. BS: balanced crystalloids; NS: normal saline.

**FIGURE 5:**
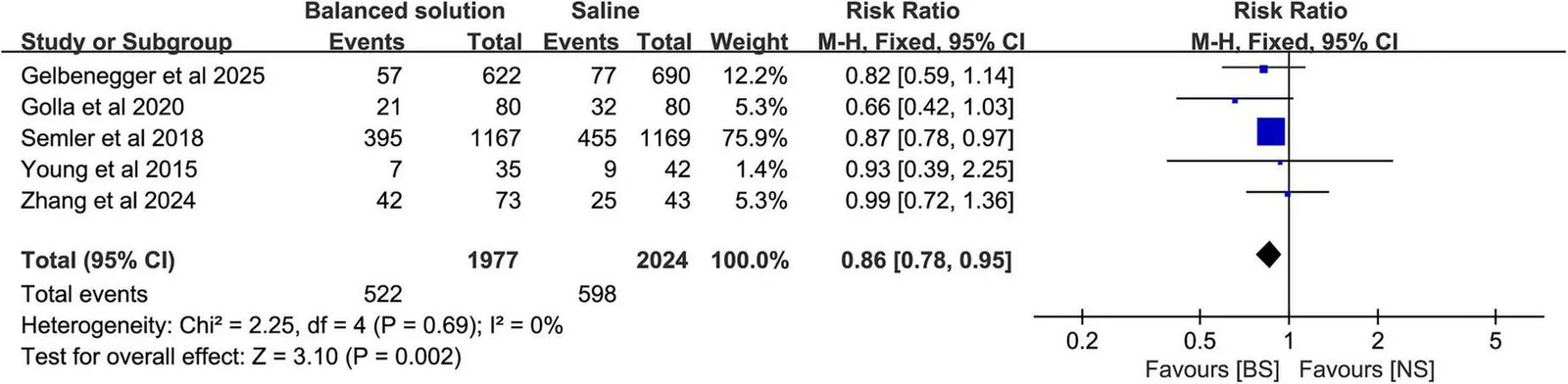
Sensitivity analysis for acute kidney injury (AKI) including the *post-hoc* observational data. BS, balanced crystalloids; NS, normal saline.

A total of five studies reported the incidence of acute kidney injury (15, 23, 24, 28, 30), among these, four were RCTs (15, 24, 28, 30) and one was a *post-hoc* observational analysis (23). In the primary analysis pooling the four RCTs, the AKI incidence was 39.1% (521/1334) in the NS group and 34.3% (465/1355) in the BS group, corresponding to a significant reduction with balanced crystalloids (RR 0.86; 95% CI 0.78–0.96; *P* = 0.004; I^2^ = 0%) (Figure 4). In a sensitivity analysis that additionally included the observational data (23), the estimate was similar (RR 0.86; 95% CI 0.78–0.95; *P* = 0.002; I^2^ = 0%) (Figure 5).

### Hyperchloremia

**FIGURE 6:**
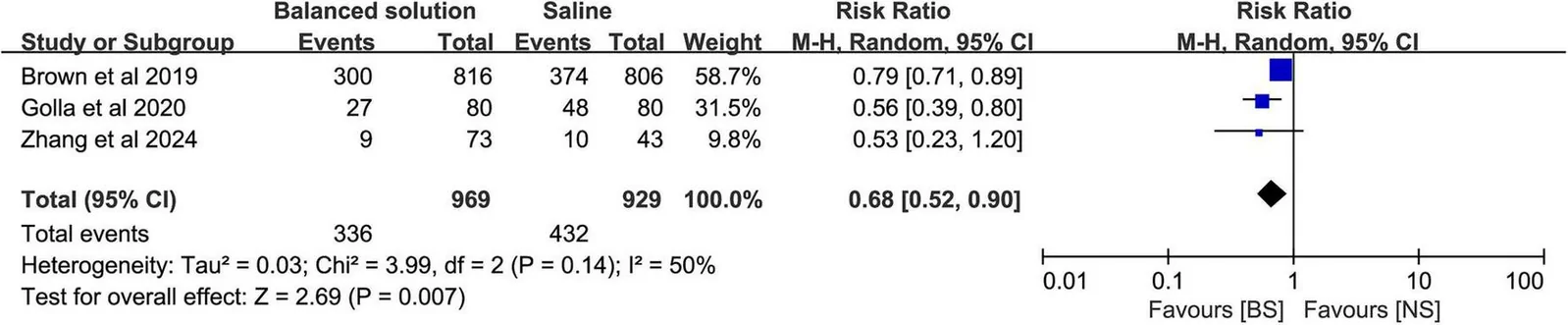
Forest plot displaying the incidence of hyperchloremia.

Three studies reported the incidence of hyperchloremia (24, 28, 29). In the Brown et al. study (29), a total of 8 patients in the BS group and 12 patients in the NS group did not have a chloride measured after enrollment. The heterogeneity test revealed significant heterogeneity (*p* < 0.05, I^2^ = 50%). A random-effects model was employed for meta-analysis. The hyperchloremia incidence in the NS group was 46.5% (432/929), while that in the BS group was 34.6% (336/969). Compared with the NS group, the BS group had a significantly lower incidence of hyperchloremia (RR 0.68; 95% CI 0.52–0.90; *P* = 0.007) (Figure 6). The high heterogeneity reduces the credibility of these findings.

### Renal replacement therapy

**FIGURE 7:**
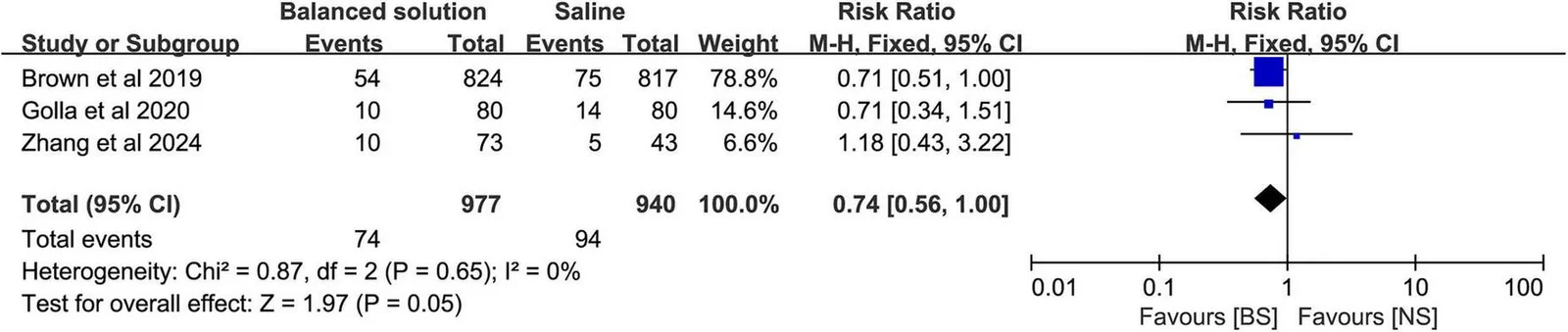
Forest plot displaying the requirement for renal replacement therapy.

Three studies involving 1,917 patients reported the requirement for RRT (24, 28, 29), with rates of 10% (94/940) in the NS group and 7.6% (74/977) in the BS group. Compared with the NS group, the BS group demonstrated a trend lower renal replacement therapy utilization (RR 0.74; 95% CI 0.56–1.00; *P* = 0.05; Heterogeneity I^2^ = 0%) (Figure 7).

### ICU-free days

**FIGURE 8:**
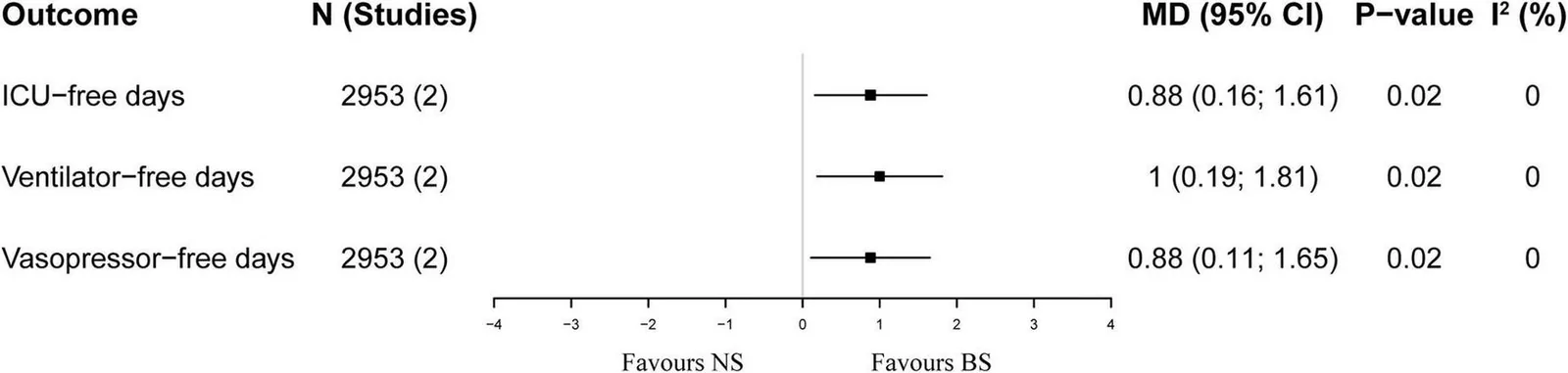
Summary forest plot for secondary outcomes of patients with sepsis or septic shock. BS indicates balanced solutions; CI, confidence interval.

Two studies (23, 29) involving 2,953 patients reported ICU-free days within 28 days. Patients in the BS group experienced longer ICU-free days compared to the NS group (MD = 0.88, 95% CI 0.16- 1.61, *P* = 0.02, Heterogeneity I^2^ = 0%) (Figure 8 and Supplementary Figure 2).

### Ventilator-free days

Two studies (23, 29) including 2,953 patients reported ventilator-free days. Compared with the NS group, patients in the BS group experienced longer ventilator-free days (MD = 1.0, 95% CI 0.19–1.81, *P* = 0.02, heterogeneity I^2^ = 0%) (Figure 8 and Supplementary Figure 3).

### Vasopressor-free days

Two studies (23, 29) including 2,953 patients reported vasopressor-free days. Compared with the NS group, patients in the BS group experienced longer vasopressor-free days (MD = 0.88, 95% CI 0.11–1.65, *P* = 0.02, heterogeneity I^2^ = 0%) (Figure 8 and Supplementary Figure 4).

### Grading the evidence

The summary of findings, including the certainty of evidence for each outcome, is presented in Supplementary Table 2. The certainty of evidence was rated as moderate for mortality and AKI; low for ICU-free days, ventilator-free days, vasopressor-free days, and RRT; and very low for hyperchloremia.

## Discussion

This meta-analysis incorporated eight clinical trials, including five multicenter and three single-center studies, totaling 8173 sepsis patients, to compare the efficacy of balanced crystalloid solutions versus normal saline in fluid resuscitation. The results demonstrated that balanced crystalloid solutions significantly reduced mortality, the incidence of AKI, the occurrence of hyperchloremia, and the requirement for RRT in sepsis patients compared to normal saline. Additionally, patients receiving balanced crystalloids experienced longer ventilator-free days, vasopressor-free days, and ICU-free days. These results further demonstrated the advantages of balanced crystalloid solutions in fluid resuscitation for sepsis, particularly in the context of the 2026 Surviving Sepsis Campaign Guidelines conditional recommendation (with low-quality evidence) for the use of balanced crystalloid (4). This study provides additional clinical evidence supporting this recommendation.

The findings of this study indicated that balanced solutions can reduce the mortality rate in sepsis patients, aligning with the overall trend observed in multiple recent studies. A retrospective cohort study involving 53,448 patients with sepsis revealed that balanced solutions were associated with a lower mortality rate (19.6% vs. 22.8%) (7). The meta-analysis published by Chen and Gao in 2023 (including 36,224 critically ill patients) similarly demonstrated that balanced solutions reduce composite mortality, with a more pronounced effect in the sepsis subgroup (31). Additionally, a network meta-analysis published in 2025 ranked balanced solutions as the preferred resuscitation fluid for septic shock, with SUCRA values (surface under the cumulative ranking curve) as high as 83.1% (32). The difference in mortality rates between the BS group and the NS group may be explained by hyperchloremia induced by saline use. It is well-established that the chloride ion concentration in saline significantly exceeds physiological plasma levels. Substantial infusion readily induces hyperchloremia, which can subsequently lead to a series of pathological changes, including metabolic acidosis, ultimately impacting patient prognosis (33). In contrast, the chloride ion concentration in balanced solutions approximates physiological levels. This distinction forms the pathophysiological basis for the potential survival benefit associated with balanced solutions. The findings of this study indicated a higher incidence of hyperchloremia in the NS group compared to the BS group. Research has found that worsening hyperchloremia in critically ill patients is associated with an increased all-cause mortality rate (34, 35). On the other hand, hyperchloremia contributes to inadequate renal perfusion and subsequently triggers acute kidney injury (36, 37). And the research indicated that AKI significantly increases the mortality rate in critically ill patients (38, 39).

The kidney, being among the first organs affected in sepsis, experiences functional deterioration that not only directly elevates the risk of AKI and RRT but also propagates a vicious cycle of multi-organ dysfunction through mechanisms including fluid overload, accumulation of metabolic waste products, and impaired clearance of inflammatory mediators. The findings of this study indicated that compared to the NS group, the BS group had a lower incidence of AKI. The mechanism by which large-volume saline infusion induces AKI involves chloride ion influx through macula densa NKCC2 channels, triggering tubuloglomerular feedback via adenosine- and thromboxane-mediated signaling pathways, leading to afferent arteriolar contraction and decreased glomerular filtration rate (6, 40–42). Concurrently, reduced renal blood flow activates the intrarenal renin-angiotensin system, further exacerbating afferent arteriolar constriction and sodium and water retention (6). Another important factor is that patients with sepsis are more susceptible to the effects of saline compared to other critically ill patients. On the one hand, sepsis itself carries a risk of lactic acidosis; a high chloride load increases the anion gap, exacerbating the acidosis. On the other hand, the systemic inflammatory response and microcirculatory disturbances caused by sepsis render the kidneys more vulnerable to additional injurious factors. The study by Suetrong et al. reported that in patients with severe sepsis and septic shock, even a moderate elevation in serum chloride ion levels was significantly associated with the occurrence of AKI (43). A large prospective study confirmed that implementing a chloride-restricted fluid therapy strategy in the intensive care unit reduces the incidence of acute kidney injury (44).

Renal replacement therapy is an important supportive measure for patients with sepsis-associated AKI; approximately 15%–20% of patients with sepsis-associated AKI require RRT (45). The requirement for RRT is not only a marker of the severity of kidney injury but is also closely associated with healthcare resource utilization and poor prognosis. This study found that compared with the NS group, the BS group had a lower requirement for RRT (7.6% vs. 10%, *P* = 0.05). Results from the SMART trial reported that balanced solutions reduced composite kidney adverse events, including RRT (29). Another meta-analysis demonstrated that balanced solutions decreased the need for RRT in sepsis patients. Furthermore, compared with resuscitation strategies such as saline and hydroxyethyl starch, balanced solutions achieved the highest SUCRA value (75.5%), suggesting it as the optimal approach for sepsis fluid resuscitation (32). Additionally, some studies have indicated that balanced solutions did not reduce the requirement for RRT in sepsis patients (26, 46). The reasons for these discrepancies include differences in the included populations, such as the SMART trial focusing on subgroup analysis of sepsis patients, whereas the BASICS trial enrolled a broader critically ill population, as well as variations in the volume of fluid administered. In conclusion, this calls for conducting large-scale RCTs specifically targeting sepsis patients to clarify the advantages of balanced solutions regarding renal replacement therapy requirements.

Our adult findings differ from the recent PRoMPT BOLUS trial in pediatric septic shock (*N* = 9,041), which found no difference in MAKE30 or 90-day mortality (2.3% vs. 2.1%) between balanced fluids and saline (47). Despite biochemical benefits with balanced fluids, these did not translate into clinical improvement. This discrepancy may be explained by lower baseline event rates, greater physiological reserve, and fewer comorbidities in children. Notably, point estimates favored balanced fluids in the sickest pediatric subgroups, suggesting that severity-dependent effects may exist across age groups. These findings underscore that adult sepsis trial results cannot be directly extrapolated to children, and highlight the need for population-specific evidence.

This study found that patients in the balanced solutions group had more ICU-free days, more ventilator-free days, and more vasopressor-free days. It should be noted that for these outcome measures, the number of studies included in the analysis was limited, and the results must be interpreted with caution, as the pooled estimates were derived from one RCT and one *post-hoc* observational analysis. Our results are consistent with a secondary analysis of the SMART trial (29) and a recent meta-analysis (32), both of which reported benefits of balanced crystalloids on organ support-free days and hospital length of stay. These findings suggest balanced solutions not only improve survival and renal outcomes but may also accelerate organ function recovery, reducing the need for intensive care resources. By reducing hyperchloremia and metabolic acidosis, balanced solutions help maintain cardiovascular function and respiratory muscle performance, consequently decreasing dependence on vasoactive agents and mechanical ventilation (48, 49); meanwhile, improvement in renal function also reduced the prolongation of hospital stay resulting from complications such as fluid overload and electrolyte imbalances (39). These patient-centered endpoints further substantiate the value of balanced solutions in the comprehensive management of sepsis, demonstrating their positive impact on clinical resource utilization and patient quality of life.

Although incorporating the latest research for thorough analysis, this study has the following limitations: ① Varying definitions of the study population, with differences in sepsis definitions (Sepsis-2 vs. Sepsis-3) across studies; some studies included patients with septic shock. ② Heterogeneity existed in the types of balanced solutions (Ringer’s lactate and Plasma-Lyte differ in buffer systems and calcium content) and total fluid volumes administered, potentially affecting the precision of effect estimates. Currently, there are studies comparing whether the differences in the composition of different balanced solutions will affect the treatment outcomes of critically ill patients (50); ③ Variations in pre-enrollment treatment existed, as some studies did not account for fluid types administered before enrollment. Some included patients had received both saline and balanced solutions prior to enrollment, which may impact the precision of effect estimates; ④ Long-term outcomes (such as progression from AKI to chronic kidney disease and long-term quality of life) were not assessed. ⑤ Most included trials were not specifically designed for sepsis populations but reported sepsis subgroup analyses; therefore, baseline imbalances cannot be entirely excluded.

## Conclusion

This systematic review and meta-analysis indicated that for fluid resuscitation in patients with sepsis, balanced solutions compared with saline significantly reduce mortality rates and kidney-related adverse events, better maintain electrolyte homeostasis, and reduced ICU length of stay, duration of vasoactive agent use, and mechanical ventilation time. Balanced solutions may be considered the preferred crystalloid solution for fluid resuscitation in patients with sepsis. Future research should focus on specific subgroups, long-term outcomes, and individualized strategies to optimize clinical practice.

## Data Availability

The original contributions presented in this study are included in this article/Supplementary material, further inquiries can be directed to the corresponding author.

## References

[1] Rudd KE Johnson SC Agesa KM Shackelford KA Tsoi D Kievlan DR et al. Global, regional, and national sepsis incidence and mortality, 1990–2017: analysis for the global burden of disease. *Lancet.* (2020) 395:200–11. 10.1016/s0140-6736(19)32989-7 31954465 PMC6970225

[2] Mok G Hendin A Reardon P Hickey M Gray S Yadav K. Macrocirculatory and microcirculatory endpoints in sepsis resuscitation. *J Intensive Care Med*. (2021) 36:1385–91. 10.1177/0885066620982585 33375916

[3] Rhee C Chiotos K Cosgrove SE Heil EL Kadri SS Kalil AC et al. Infectious diseases society of america position paper: recommended revisions to the national severe sepsis and septic shock early management bundle (SEP-1) sepsis quality measure. *Clin Infect Dis*. (2021) 72:541–52. 10.1093/cid/ciaa059 32374861 PMC8189682

[4] Prescott HC Antonelli N Alhazzani W Møller MH Alshamsi F Azevedo LCP et al. Surviving sepsis campaign: international guidelines for management of sepsis and septic shock 2026. *Crit Care Med.* (2026) 54:725–812. 10.1097/CCM.0000000000007075 41869847

[5] Finfer S Liu B Taylor C Bellomo R Billot L Cook D et al. Resuscitation fluid use in critically ill adults: an international cross-sectional study in 391 intensive care units. *Crit Care*. (2010) 14:R185. 10.1186/cc9293 20950434 PMC3219291

[6] Chowdhury AH Cox EF Francis ST Lobo DN. A randomized, controlled, double-blind crossover study on the effects of 2-L infusions of 0.9% saline and plasma-lyte^®^ 148 on renal blood flow velocity and renal cortical tissue perfusion in healthy volunteers. *Ann Surg*. (2012) 256:18–24. 10.1097/SLA.0b013e318256be72 22580944

[7] Raghunathan K Shaw A Nathanson B Stürmer T Brookhart A Stefan MS et al. Association between the choice of IV crystalloid and in-hospital mortality among critically ill adults with sepsis*. *Crit Care Med*. (2014) 42:1585–91. 10.1097/CCM.0000000000000305 24674927

[8] Kellum JA Song M Almasri E. Hyperchloremic acidosis increases circulating inflammatory molecules in experimental sepsis. *Chest*. (2006) 130:962–7. 10.1378/chest.130.4.962 17035425

[9] Kellum JA Song M Venkataraman R. Effects of hyperchloremic acidosis on arterial pressure and circulating inflammatory molecules in experimental sepsis. *Chest*. (2004) 125:243–8. 10.1378/chest.125.1.243 14718447

[10] Passmore MR Byrne L Obonyo NG See Hoe LE Boon AC Diab SD et al. Inflammation and lung injury in an ovine model of fluid resuscitated endotoxemic shock. *Respir Res*. (2018) 19:231. 10.1186/s12931-018-0935-4 30466423 PMC6249903

[11] Langer T Santini A Scotti E Van Regenmortel N Malbrain ML Caironi P. Intravenous balanced solutions: from physiology to clinical evidence. *Anaesthesiol Intensive Ther.* (2015) 47:s78–88. 10.5603/AIT.a2015.0079 26588483

[12] Carvalho Pereira L Carvalho Pereira I Delfino Cabral T Viana P Ribeiro A Amaral S. Balanced crystalloids versus normal saline in kidney transplant patients: an updated systematic review, meta-analysis, and trial sequential analysis. *Anesth Analg*. (2024) 139:58–67. 10.1213/ANE.0000000000006932 38578867

[13] Yunos NM Kim IB Bellomo R Bailey M Ho L Story D et al. The biochemical effects of restricting chloride-rich fluids in intensive care. *Crit Care Med*. (2011) 39:2419–24. 10.1097/CCM.0b013e31822571e5 21705897

[14] Wilkes NJ Woolf R Mutch M Mallett SV Peachey T Stephens R et al. The effects of balanced versus saline-based hetastarch and crystalloid solutions on acid-base and electrolyte status and gastric mucosal perfusion in elderly surgical patients. *Anesth Analg*. (2001) 93:811–6. 10.1097/00000539-200110000-00003 11574338

[15] Semler MW Self WH Wanderer JP Ehrenfeld JM Wang L Byrne DW et al. Balanced crystalloids versus saline in critically Ill adults. *N Engl J Med.* (2018) 378:829–39. 10.1056/NEJMoa1711584 29485925 PMC5846085

[16] Patel JJ Jasti J Hu K. Lactated ringer’s or normal saline for initial fluid resuscitation in sepsis-induced hypotension. *Crit Care Med*. (2025) 53:e2120–1. 10.1097/CCM.0000000000006764 41055445

[17] Hammond NE Zampieri FG Di Tanna GL Garside T Adigbli D Cavalcanti AB et al. Balanced crystalloids versus saline in critically Ill Adults - a systematic review with meta-analysis. *NEJM Evid*. (2022) 1:EVIDoa2100010. 10.1056/EVIDoa2100010 38319180

[18] Beran A Altorok N Srour O Malhas SE Khokher W Mhanna M et al. Balanced crystalloids versus normal saline in adults with sepsis: a comprehensive systematic review and meta-analysis. *J Clin Med*. (2022) 11:1971. 10.3390/jcm11071971 35407578 PMC8999853

[19] Higgins JP Altman DG Gøtzsche PC Jüni P Moher D Oxman AD et al. The cochrane collaboration’s tool for assessing risk of bias in randomised trials. *BMJ*. (2011) 343:d5928. 10.1136/bmj.d5928 22008217 PMC3196245

[20] Kellum JA Lameire N Aspelin P Barsoum RS Burdmann EA Goldstein SL et al. Kidney disease: improving global outcomes (KDIGO) acute kidney injury work group. *Nephron Clin Pract.* (2012) 120:c179–84. 10.1159/000339789 22890468

[21] Luo D Wan X Liu J Tong T. Optimally estimating the sample mean from the sample size, median, mid-range, and/or mid-quartile range. *Stat Methods Med Res*. (2018) 27:1785–805. 10.1177/0962280216669183 27683581

[22] Guyatt GH Oxman AD Vist GE Kunz R Falck-Ytter Y Alonso-Coello P et al. GRADE: an emerging consensus on rating quality of evidence and strength of recommendations. *BMJ*. (2008) 336:924–6. 10.1136/bmj.39489.470347.AD 18436948 PMC2335261

[23] Gelbenegger G Shapiro NI Zeitlinger M Jilma B Douglas IS Jorda A. Lactated ringer’s or normal saline for initial fluid resuscitation in sepsis-induced hypotension. *Crit Care Med*. (2025) 53:e1140–4. 10.1097/CCM.0000000000006601 39969246 PMC12047640

[24] Zhang J Liu F Wu Z Jiang J Wang B Qian Y et al. Acetate ringer’s solution versus normal saline solution in sepsis: a randomized. controlled trial. *Shock*. (2024) 61:520–6. 10.1097/SHK.0000000000002324 38369528

[25] Finfer S Micallef S Hammond N Navarra L Bellomo R Billot L et al. Balanced multielectrolyte solution versus saline in critically Ill adults. *N Engl J Med*. (2022) 386:815–26. 10.1056/NEJMoa2114464 35041780

[26] Zampieri FG Machado FR Biondi RS Freitas FGR Veiga VC Figueiredo RC et al. Effect of intravenous fluid treatment with a balanced solution vs 0.9% saline solution on mortality in critically ill patients. *JAMA*. (2021) 326:818. 10.1001/jama.2021.11684 34375394 PMC8356144

[27] Pagano A Porta G Bosso G Rosato V. Ringer lactate versus saline solution for resuscitation of sepsis and septic shock. *Ital. J. Emerg.* (2020) 9:29–34.

[28] Golla R Kumar S Dhibhar DP Bhalla A Sharma N. 0.9% saline V/S Ringer’s lactate for fluid resuscitation in adult sepsis patients in emergency medical services: an open-label randomized controlled trial. *Hong Kong J Emerg Med*. (2022) 29:271–80. 10.1177/1024907920948983

[29] Brown RM Wang L Coston TD Krishnan NI Casey JD Wanderer JP et al. Balanced crystalloids versus saline in sepsis. *Am J Respir Crit Care Med*. (2019) 200:1487–95. 10.1164/rccm.201903-0557OC 31454263 PMC6909845

[30] Young P Bailey M Beasley R Henderson S Mackle D McArthur C et al. Effect of a buffered crystalloid solution vs saline on acute kidney injury among patients in the intensive care unit: the split randomized clinical trial. *JAMA*. (2015) 314:1701–10. 10.1001/jama.2015.12334 26444692

[31] Chen Y Gao Y. Comparison of balanced crystalloids versus normal saline in critically Ill patients: a systematic review with meta-analysis and trial sequential analysis of randomized controlled trials. *Ther Clin Risk Manag*. (2023) 19:783–99. 10.2147/TCRM.S416785 37850070 PMC10577264

[32] Song B Fu K Zheng X Liu C. Fluid resuscitation in adults with severe infection and sepsis: a systematic review and network meta-analysis. *Front Med.* . (2025) 12:1543586. 10.3389/fmed.2025.1543586 40600034 PMC12209188

[33] Finfer S Myburgh J Bellomo R. Intravenous fluid therapy in critically Ill adults. *Nat Rev Nephrol.* (2018) 14:541–57. 10.1038/s41581-018-0044-0 30072710

[34] Neyra JA Canepa-Escaro F Li X Manllo J Adams-Huet B Yee J et al. Association of hyperchloremia with hospital mortality in critically Ill septic patients. *Crit Care Med*. (2015) 43:1938–44. 10.1097/CCM.0000000000001161 26154934 PMC4537691

[35] Shad ZS Qureshi MSS Qadeer A Abdullah A Munawar K Khan MT et al. Hyperchloremia in intensive care unit mortality: an underestimated fact. *Cureus*. (2019) 11:e4770. 10.7759/cureus.4770 31363451 PMC6663040

[36] Kim SY Huh KH Lee JR Kim SH Jeong SH Choi YS. Comparison of the effects of normal saline versus plasmalyte on acid-base balance during living donor kidney transplantation using the Stewart and base excess methods. *Transplant Proc*. (2013) 45:2191–6. 10.1016/j.transproceed.2013.02.124 23953528

[37] Hansen PB Jensen BL Skott O. Chloride regulates afferent arteriolar contraction in response to depolarization. *Hypertension*. (1998) 32:1066–70. 10.1161/01.hyp.32.6.1066 9856975

[38] Mandelbaum T Lee J Scott DJ Mark RG Malhotra A Howell MD et al. Empirical relationships among oliguria, creatinine, mortality, and renal replacement therapy in the critically Ill. *Intensive Care Med*. (2013) 39:414–9. 10.1007/s00134-012-2767-x 23223822 PMC3580030

[39] Chertow GM Burdick E Honour M Bonventre JV Bates DW. Acute kidney injury, mortality, length of stay, and costs in hospitalized patients. *J Am Soc Nephrol*. (2005) 16:3365–70. 10.1681/ASN.2004090740 16177006

[40] Malbrain MLNG Langer T Annane D Gattinoni L Elbers P Hahn RG et al. Intravenous fluid therapy in the perioperative and critical care setting: executive summary of the international fluid academy (IFA). *Ann Intensive Care*. (2020) 10:64. 10.1186/s13613-020-00679-3 32449147 PMC7245999

[41] Bullivant EM Wilcox CS Welch WJ. Intrarenal vasoconstriction during hyperchloremia: role of thromboxane. *Am J Physiol*. (1989) 256:F152–7. 10.1152/ajprenal.1989.256.1.F152 2912160

[42] Wilcox CS. Regulation of renal blood flow by plasma chloride. *J Clin Invest*. (1983) 71:726–35. 10.1172/jci110820 6826732 PMC436923

[43] Suetrong B Pisitsak C Boyd JH Russell JA Walley KR. Hyperchloremia and moderate increase in serum chloride are associated with acute kidney injury in severe sepsis and septic shock patients. *Crit Care*. (2016) 20:315. 10.1186/s13054-016-1499-7 27716310 PMC5053142

[44] Shaw AD Schermer CR Lobo DN Munson SH Khangulov V Hayashida DK et al. Impact of intravenous fluid composition on outcomes in patients with systemic inflammatory response syndrome. *Crit Care*. (2015) 19:334. 10.1186/s13054-015-1045-z 26370823 PMC4570151

[45] Lameire N Vanmassenhove J. Timing of dialysis in sepsis and acute respiratory distress syndrome. *Am J Respir Crit Care Med*. (2018) 198:4–5. 10.1164/rccm.201801-0129ED 29394089

[46] Liu C Lu G Wang D Lei Y Mao Z Hu P et al. Balanced crystalloids versus normal saline for fluid resuscitation in critically ill patients: a systematic review and meta-analysis with trial sequential analysis. *Am J Emerg Med*. (2019) 37:2072–8. 10.1016/j.ajem.2019.02.045 30852043

[47] Balamuth F Weiss SL Long E Thompson GC Artis AS Campos ML et al. Bolus Investigators of the pecarn, and p. networks, balanced fluid or 0.9% saline in children treated for septic shock. *N Engl J Med.* (2026). 10.1056/NEJMoa2601969 [Epub ahead of print]. 42028918 PMC13134814

[48] Sudheer N James JV. Fluid management in critical care: new insights into optimal fluid therapy. *Cureus*. (2024) 16:e75436. 10.7759/cureus.75436 39791089 PMC11712523

[49] Pfortmueller CA Funk GC Reiterer C Schrott A Zotti O Kabon B et al. Normal saline versus a balanced crystalloid for goal-directed perioperative fluid therapy in major abdominal surgery: a double-blind randomised controlled study. *Br J Anaesth*. (2018) 120:274–83. 10.1016/j.bja.2017.11.088 29406176

[50] Qian ET Brown RM Jackson KE Wang L Stollings JL Freundlich RE et al. Normosol-R vs lactated ringers in the critically Ill: a randomized trial. *Chest*. (2025) 168:336–45. 10.1016/j.chest.2025.02.008 39971001 PMC12405922

