# Supplementary material for: Balanced crystalloid versus saline for resuscitation in adult sepsis: a systematic review and meta-analysis
Source: Front Med (Lausanne). 2026 Aug 21;13:1906761. doi: 10.3389/fmed.2026.1906761 (PMC13541683; doi:10.3389/fmed.2026.1906761)
Supplement: Supplementary file 1 [file Supplementary_file_1.docx]

Supplementary table 1. Baseline characteristics of included studies

| Study  Author year | Group | Age, yr  Mean（SD） | Gender, male  (N/total) | SOFA score  Mean（SD） | Volume of intravenous NS in the 24 hours prior to enrollment | Volume of intravenous BS in the 24 hours prior to enrollment | Volume of intravenous NS in the 24 hours after enrollment | Volume of intravenous NS in the 24 hours after enrollment |
| --- | --- | --- | --- | --- | --- | --- | --- | --- |
| Young et al (2015) £ | BS | 60.1(16.79) | 739/1152 | NR | 551 ± 1157ml | 1748 ± 1964ml | 1711±1385ml | 0±0ml |
|  | NS | 60.95(16.25) | 746/1110 | NR | 549 ± 1098ml | 1649 ± 1841ml | 1±45ml | 1694±1292ml |
| Semler et al (2018) £ | BS | 58(44,69) ^⨍^ | 4540/7942 | NR | 125±465ml | 790±1329ml | NR | NR |
|  | NS | 58(44,69) ^⨍^ | 4557/7860 | NR | 400±896ml | 476±1073 ml | NR | NR |
| Brown et al (2019) * | BS | 60(48,69) ^⨍^ | 451/824 | 7(5,10) ^⨍^ | NR | NR | NR | NR |
|  | NS | 60(60,47) ^⨍^ | 448/817 | 8(5,11) ^⨍^ | NR | NR | NR | NR |
| Golla et al (2020) | BS | 43.46(17.99) | 45/80 | 7.64(2.56) | NR | NR | NR | 3.74±0.9(L) |
|  | NS | 42.44(19.37) | 40/80 | 7.63(2.49) | NR | NR | 33.66±0.79(L) | NR |
| Pagano et al (2020) | BS | 75.9(12.3) | 26/35 | 5.9(2.9) | NR | NR | NR | NR |
|  | NS | 75.8(12.1) | 25/49 | 6(2.8) | NR | NR | NR | NR |
| Zampieri et al (2021) | BS | 60.9(17) ^£^ | 2909/5230 ^£^ | 4(2,7) ^⨍ £^ | 0（0-1000）ml ^⨍^ | 0(0-1500) ml ^⨍^ | NR | 1500(900-2087.5) ml ^⨍^ |
|  | NS | 61.2(16.9) ^£^ | 2956/5290 ^£^ | 4(2,7) ^⨍ £^ | 0（0-1000）ml ^⨍^ | 0(0-1500) ml ^⨍^ | 1500(919.5-2126) ml ^⨍^ | NR |
| Finfer et al (2022) | BS | 61.7(16.4) ^£^ | 1578/2515 ^£^ | 3(1,4) ^⨍ £^ | 1060.5±1280.83 ml | 606.1±1505.64 | NR | NR |
|  | NS | 62.1(16.9) ^£^ | 1511/2522 ^£^ | 3(1,4) ^⨍ £^ | 1006.7±1211.31ml | 655.9±1590.54 | NR | NR |
| Zhang et al (2024) | BS | 63(12.1) | 52/73 | 7(5,8) ^⨍^ | NR | NR | NR | NR |
|  | NS | 62.3(12.5) | 25/43 | 8(6,10) ^⨍^ | NR | NR | NR | NR |
| Gelbenegger et al (2025) | BS | 57.34(15.65) | 331/622 | 3(1,5) | 0.5±7ml | 1673±558ml | 828.0±1270ml | 3249±1495ml ^€^ |
|  | NS | 60.67(15.5) | 364/690 | 3(1,5) | 1737±599ml | 0.32±4.1ml | 2635±1308ml | 278±676ml ^€^ |

Note: &: Range, ⨍: Median (IQR); *: These two articles originating from the same clinical trial but reporting different outcome measures; hence, two publications were included for specific outcome analyses. £: Data in the entire study population, not just in patients with sepsis. €: The volume administered within 24 hours, including pre-randomization period. SOFA: Sequential organ failure assessment. BS: balanced solutions group. NS: saline group.

Supplementary table 2. GRADE summary of findings table

| **Number of studies** | **Number of patients** | | **Effect** | | **Certainty of evidence**  **(GRADE)** |
| --- | --- | --- | --- | --- | --- |
|  | **BS** | **NS** | **Relative (95% CI)** | **Absolute (95% CI)** |  |
| **Mortality** | | | | | |
| 8 | 1164/4056 (28.7%) | 1302/4117 (31.6%) | **RR 0.91 (0.85 to 0.97)** | **28 fewer per 1,000**  (from 47 fewer to 9 fewer) | ⨁⨁⨁◯ Moderate ^a^ |
| **The incidence of AKI** | | | | | |
| 5 | 522/1977 (26.4%) | 598/2024 (29.5%) | **RR 0.86 (0.78 to 0.95)** | **41 fewer per 1,000**  (from 65 fewer to 15 fewer) | ⨁⨁⨁◯  Moderate ^a^ |
| **Hyperchloremia** | | | | | |
| 3 | 336/969 (34.7%) | 432/929 (46.5%) | **RR 0.68 (0.52 to 0.90)** | **149 fewer per 1,000**  (from 223 fewer to 47 fewer) | ⨁◯◯◯  Very low ^a, b, c^ |
| **Renal replacement therapy** | | | | | |
| 3 | 74/977 (7.6%) | 94/940 (10.0%) | **RR 0.74 (0.56 to 1.00)** | **26 fewer per 1,000**  (from 44 fewer to 0 fewer) | ⨁⨁◯◯  Low ^a, d^ |
| **ICU-free days** | | | | | |
| 2 | 1446 | 1507 | - | **MD 0.88 higher**  (0.16 higher to 1.61 higher) | ⨁⨁◯◯  Low ^e^ |
| **Ventilator-free days** | | | | | |
| 2 | 1446 | 1507 | - | **MD 1 higher**  (0.19 higher to 1.81 higher) | ⨁⨁◯◯  Low ^e^ |
| **Vasopressor-free days** | | | | | |
| 2 | 1446 | 1507 | - | **MD 0.88 higher**  (0.11 higher to 1.65 higher) | ⨁⨁◯◯  Low ^e^ |

Note: CI: confidence interval; MD: mean difference; RR: risk ratio. a. Among the included studies, some explicitly did not implement blinding or employed an open-label design, presenting a potential risk of selective reporting or outcome detection bias. b. In the Brown et al. study, a total of 8 patients in the BS group and 12 patients in the NS group did not have a chloride measured after enrollment. c. I² statistics indicate the possible presence of moderate heterogeneity. d. The upper limit of the confidence interval exactly touches 1.00 (the threshold for no effect), meaning the result is simultaneously compatible with both "significant benefit" (RR=0.56) and "no effect" (RR=1.00). e. This pooled estimate included one RCT and one observational study. Per GRADE, non‑randomized evidence starts as low; no further downgrading was applied (I² = 0%; precise CI; no indirectness or publication bias). Overall certainty: low. BS: balanced solutions group. NS: saline group.

Supplementary figure 1. Risk assessment graph according to the Cochrane risk assessment tool.

Supplementary figure 2. Meta-analysis and forest plot showed the ICU-free days. BS: Balanced solutions group. NS: Normal saline group.

Supplementary figure 3. Meta-analysis and forest plot showed the ventilator-free days. BS: Balanced solutions group. NS: Normal saline group.

Supplementary figure 4. Meta-analysis and forest plot showed the vasopressor-free days. BS: Balanced solutions group. NS: Normal saline group.
